# Caregiving burnout of community-dwelling people with dementia in Hong Kong and New Zealand: a cross-sectional study

**DOI:** 10.1186/s12877-021-02153-6

**Published:** 2021-04-20

**Authors:** Crystal Y. Chan, Gary Cheung, Adrian Martinez-Ruiz, Patsy Y. K. Chau, Kailu Wang, E. K. Yeoh, Eliza L. Y. Wong

**Affiliations:** 1 Jockey Club School of Public Health and Primary Care, The Chinese University of Hong Kong, Hong Kong, China; 2 Department of Psychological Medicine, University of Auckland, Auckland, New Zealand; 3 Instituto Nacional De Geriatría, Mexico City, Mexico; 4 Department of Population Health, University of Auckland, Auckland, New Zealand

**Keywords:** Informal caregiver, Caregiver burnout, Caregiver stress, Community-dwelling older adult, Dementia, Ageing-in-place

## Abstract

**Background:**

Informal caregiving for people with dementia can negatively impact caregivers’ health. In Asia-Pacific regions, growing dementia incidence has made caregiver burnout a pressing public health issue. A cross-sectional study with a representative sample helps to understand how caregivers experience burnout throughout this region. We explored the prevalence and contributing factors of burnout of caregivers of community-dwelling older people with dementia in Hong Kong (HK), China, and New Zealand (NZ) in this study.

**Methods:**

Analysis of interRAI Home Care Assessment data for care-recipients (aged ≥65 with Alzheimer’s disease/other dementia) who had applied for government-funded community services and their caregivers was conducted. The sample comprised 9976 predominately Chinese in HK and 16,725 predominantly European in NZ from 2013 to 2016. Caregiver burnout rates for HK and NZ were calculated. Logistic regression was used to determine the adjusted odds ratio (AOR) of the significant factors associated with caregiver burnout in both regions.

**Results:**

Caregiver burnout was present in 15.5 and 13.9% of the sample in HK and NZ respectively. Cross-regional differences in contributing factors to burnout were found. Care-recipients’ ADL dependency, fall history, and cohabitation with primary caregiver were significant contributing factors in NZ, while primary caregiver being child was found to be significant in HK. Some common contributing factors were observed in both regions, including care-recipients having behavioural problem, primary caregiver being spouse, providing activities-of-daily-living (ADL) care, and delivering more than 21 h of care every week. In HK, allied-health services (physiotherapy, occupational therapy and speech therapy) protected caregiver from burnout. Interaction analysis showed that allied-health service attenuates the risk of burnout contributed by care-recipient’s older age (85+), cohabitation with child, ADL dependency, mood problem, and ADL care provision by caregivers.

**Conclusions:**

This study highlights differences in service delivery models, family structures and cultural values that may explain the cross-regional differences in dementia caregiving experience in NZ and HK. Characteristics of caregiving dyads and their allied-health service utilization are important contributing factors to caregiver burnout. A standardized needs assessment for caregivers could help policymakers and healthcare practitioners to identify caregiving dyads who are at risk of burnout and provide early intervention.

**Supplementary Information:**

The online version contains supplementary material available at 10.1186/s12877-021-02153-6.

## Background

Informal caregivers refer to unpaid workers such as relatives, friends, and neighbours who provide care to older person with dementia. These caregivers are important partners in health system and can help to ensure the principle of ageing-in-place is fulfilled. In response to the various challenges related to caregiving, caregivers may feel stressful and experience burnout as a result [1]. Caregiver burnout is defined as the stage “where the experience is no longer a viable or healthy option for either the caregiver or the person receiving care” [2], with caregivers experiencing increased stress and anxiety [3], social isolation [4], and depressive symptoms [5]. Caregiving for people with dementia is more stressful and demanding than caring for a person with a physical disability alone [6]. The changed behaviours and functional dependence associated with dementia [7] require longer durations of care, and for the caregiver to have better cognitive preparedness and physical fitness [8] which could contribute to a higher risk of burnout.

Population levels of caregiver burnout and their contributing factors are in the interests of public health researchers for improving health outcomes and health protection. In earlier explanatory studies, Perlin and his colleagues provided a stress process framework [6, 9] to illustrate the relationship between contextual factors, stressors relevant to the caregiving role and caregiver burden, which is moderated by availability of support and resources (e.g. formal services utilization and social support). Several systematic reviews have identified that these stressors, which include demographic information of the people with dementia (e.g. education [10]), their health and functional status (including cognitive functionality [11], functional status [10–13], behavioural disturbance [10, 11], neuropsychiatric symptoms [10]) and caregiver characteristics (e.g. relationship with person with dementia [13], cohabitation with care-recipients [10, 13], caregiving load [10], coping style [11]). While caregiver resources such as positive appraisal of caregiving [14] and social support [15] have been recognised as moderators between stressors and caregiver burden, the impact of formal support service utilization on the relationship between stressors and burden is rarely examined.

Ethnical and contextual influence are widely recognised to be associated with burnout, but investigation using a representative sample is limited. Ethnicity and social structure are important factors contributing to the difference in dementia caregiving experience [16–18]. For example, African American dementia caregivers were found to have better wellbeing comparing to their white counterparts [19]; while dementia caregivers in Korea is more burdened than Korean-American and white caregivers [16, 20]. Previous attempts were made to explain this difference by conducting comparison studies in regions with different ethnicity composition. In a survey of 230 Japanese and 113 Taiwanese dementia caregivers, Matsushita [11] noticed that ‘impact on caregivers’ lives’ had a larger effect on caregiver burden among Taiwanese; while Japanese caregivers were most burdened by ‘dependency of care-recipient’. These results hinted there could be cultural differences in caregivers’ evaluation of burden and perception of caregiving burden. In a mixed-method study involving 91 Chinese and 57 Australians dementia caregivers in China and Australia respectively, Xiao and colleagues [21] noted that the individualistic culture in Australia encourages caregivers to pursue for support services that tailored for their needs. The lack of tailored dementia care services resulted in a low utilization rate of community care, and mirrored as the higher level of burden in the Australian cohort [21]. These studies highlight the impact of ethnical and contextual factors on formal service utilization and the development of caregiver burden, which shed lights on the importance of developing context-specific dementia care policies. Both of these studies used a convenient sample and included a small sample size that could not produce generalizable results. Also, these studies did not investigate the similarities and differences of individual factors contributing to dementia caregiver burdens. Therefore, research that utilizes a representative population sample is needed to enrich our understandings of caregiver burnout patterns across regions of different ethnical and contextual backgrounds.

Asia anticipated the highest number of new dementia cases (3.6 million per year) among all world regions [22], and comparative research on factors contributing to caregiver burden is essential in fighting dementia across Asia-pacific countries. Despite their ethnical differences, Hong Kong Special Administrative Region of China (HK) and New Zealand (NZ) in the Asia-Pacific region face similar challenges of rising rates of dementia in their ageing populations. As a predominantly Chinese society (92%) [23], the projected number of older adults living with dementia in HK will increase to 13% of the older adult population in 2039 [24]. NZ has a predominately European population (74%) [25], with an 29% increase on the number of people diagnosed with dementia in NZ between 2011 and 2016 [26]. Since healthcare service utilization patterns differ among ethnic groups [27], this difference could impact on the presentation of dementia symptoms and the burden on caregivers.

Formal care services such as allied-health services for people living with dementia and their informal caregivers is delivered differently in HK and NZ. Service from allied-health professionals, such as physiotherapists, speech therapists and occupational therapist, are found to be effective in improving cognitive performance and daily functioning [28], and shorter hospital stay [29] of people living with dementia. Allied-health services and caregivers support services in HK are provided from piece-meal programmes at 41 District Elderly Community Centres covering different geographical areas, without a comprehensive dementia care framework. In NZ, allied health services are provided through Needs Assessment and Service Coordination Service after an interRAI assessment under the NZ Framework for Dementia Care [30]. To provide either community or residential care support for older adults, both regions offer need-based assessment, namely International Residential Assessment (InterRAI) which is a comprehensive geriatric assessment used in over 30 countries for care planning by assessing the functional status and quality of life issues of community-dwelling adults [31]. interRAI-Home Care Version (interRAI-HC) has demonstrated good inter-rater reliability in a 12-countries study [32] and its neurological assessment diagnosis has good sensitivity for identifying people with Alzheimer’s’ Diseases and other dementias [33]. interRAI is currently being used in North America (Canada and multiple states in the U.S.), Europe (Italy, Switzerland, Finland, Estonia, etc.), and Asia/Pacific Rim (HK, Singapore, Japan, Australia, NZ) and therefore can be used for comparative studies across regions. While both HK and NZ are located in the Asia-pacific region, policy supporting caregivers is at different stages in each region. Policy supporting caregivers is relatively well established in NZ, whereas similar policy in HK is only in the early development stage. The similar increasing dementia prevalence and the different political contexts between HK and NZ provides an opportunity for understanding caregiver burnout in these two Asia-Pacific regions.

The aim of this study was to investigate the proportion of caregiver burnout and explore potential contributing factors to caregiver burnout in HK and NZ as two examples of different ethnical and political contexts in the Asia-pacific regions. This study also aims to examine the impact of formal care service on the relationship between potential contributing factors and caregiver burnout. Despite the difference in caregiver policy formulation, we hypothesized that there are similarities and differences in the caregiver burnout factors pattern in Hong Kong and New Zealand. This is the first study to include all dementia caregivers whose care-recipient are applying for social care support in Hong Kong and New Zealand. By studying the contributing factors to caregiver burnout in two ethnically and political distinct regions, we not only fill the research gap but also provide evidence for health policy makers to re-design service delivery to relieve caregiver burden and support people with dementia to age well in place.

## Methods

### Subjects

This is a cross-sectional study using the routinely collected data of interRAI in HK and NZ to investigate the proportion of caregiver burnout and explore potential contributing factors to caregiver burnout as two examples of different ethnical and political contexts in the Asia-pacific regions. Data sources consisted of a population-based cohort of all older adult seeking support from public-funded community and/or residential care in HK and NZ [34, 35]. To address our research objectives, this study included a total of 26,521 older adults (HK: 9796; NZ: 16,725) who aged ≥65 years old with Alzheimer’s disease or other dementias, who had at least one informal caregiver and their first interRAI in HK and NZ from January 2013 to December 2016. All caregivers included were the primary caregiver of the person living with dementia. In HK, the SWD uses the interRAI Minimal Data Set – Home Care Assessment Version 2.0 (interRAI MDS-HC) and in NZ, DHB funded services use the interRAI Home Care Assessment version 9.1 (interRAI-HC 9.1) mandated by the Ministry of Health. Approval for data use and analysis was obtained from the Social Welfare Department of Hong Kong and the Central Region Technical Advisory Services Limited in New Zealand. A brief history of the interRAI and quality assurance mechanisms in HK and NZ could be found in S1.

### Measures

Caregiver burnout is a multi-dimensional concept, which can be measured as a composition score of subjective (e.g. emotional distress) [36, 37] and objective (e.g. physical demands and inability to care) [38] cost of caregiving. Drawing upon the available items in the secondary dataset, a previous research study [39] selected items that reflect the objective and subjective difficulties of caregiving experience, summarized the scores and grouped participants into a binary categorization (experienced burden or experience no burden). Other studies have used the binary interRAI items of “express stress” and “inability to continue” to define caregiver stress/burde n[40, 41]. With reference to our methodology, we define caregiver burnout as caregivers who (i) were distress; AND (ii) reported they were unable to continue to care for the person living with dementia to highlight the non-viable nature of caregiver burnou t[2]. Both variables assessed were binary (Yes/No) and assessed by accredited interRAI assessors at the time when the assessment was conducted. The assessments were conducted once the caregiver sough help publicly funded social services for the care-recipients in both regions. Factors that are common in both interRAI MDS-HC and interRAI-HC 9.1 were extracted and grouped into four following sections for this study: demographics information of care-recipient (sex, age, marital status, whom the care-recipient live with, and whether the care-recipient is perceived to be better-off living elsewhere); health & information status of care-recipient (whether the care-recipient stat in hospital in last 7 days, hearing ability, vision ability, IADL ability, ADL ability, fall history in 90 days, history of comorbidity, mood problem, and behavioural symptoms record); caregiver characteristics (relationship with care-recipients, whether caregiver lives with care-recipient, type of care provided by caregiver (IADL and ADL care), and whether caregiver provides more than 21 h of care in 7 days); and formal care services utilization of care-recipient (home services, visiting nurse service and allied-health services from physiotherapist, speech therapist and occupational therapist). We also included the information of ethnicity of NZ sample and the language of HK sample to give a more complete description of our study population despite the fact that they are not directly comparable. Details of variables regrouping in the two datasets was presented in S2.

### Statistical analysis

IBM Statistical Package for the Social Sciences (SPSS) Version 24 was used for statistical analysis and RStudio with R 4.0.3 was used for graphic production. Descriptive statistics of the potential factors such as means, standard deviations, frequencies and percentages were calculated to understand the characteristics of care-recipient and caregivers in HK and NZ. Caregiver burnout rate in HK and NZ was compared using chi-square test. Logistic regression was used to estimate crude odds ratio with 95% confidence interval (CI) of each factor by regions to show their association with caregiver burnout. Difference in odds ratio of individual factors between HK and NZ was tested by putting in an interaction term between the individual factors and groups in the regression model. Potential factors associated with caregiver burnout in both HK and NZ were then put into a force-entry logistic regression model. Stratified analysis and interaction term was included in the regression to examine the effect moderation of formal service utilization and the other covariates on caregiver burnout. Adjusted odds ratio (AOR) and its corresponding 95% CI of caregiver burnout from the model that is sufficient with the smallest number of degree of freedom were reported. The level of statistical significance was set at 5%.

## Results

### Prevalence of caregiver burnout

During the study period, there was a total of 26,521 respondents where 9796 from HK and 16,725 from NZ with a diagnosis of Alzheimer’s disease or other dementias were recorded in their interRAI assessment between 2013 and 2016. Prevalence of caregiver burnout was 15.5 and 13.9% in HK and NZ respectively (Table 1). The proportion of caregiver burnout was significantly higher in the group living with care-recipient in both regions (*p* < 0.001).

**Table 1:** Demographic information of living-with-dementia adults aged 65+ and their caregivers

	HK (*n* = 9796)		NZ (*n* = 16,725)	
	n (%)	Crude OR (95% CI)	n (%)	Crude OR (95% CI)
Outcome: Caregiver burnout ***	1523 (15.5)	N/A	2322 (13.9)	N/A
Caregiver feeling distress	3937 (40.2)	N/A	5265 (31.5)	N/A
Caregiver unable to continue to care	4926 (50.3)	N/A	4187 (25.1)	N/A
**Demographic Information of care-recipient**				
Female	5899 (60.2)	0.72 (0.65, 0.81)	9564 (57.2)	0.71 (0.65, 0.78)
Age				
65–74	1148 (11.7)	Ref	2764 (16.5)	Ref
75–84	4436 (45.3)	0.96 (0.81, 1.15)	7713 (46.1)	1.05 (0.92, 1.19)
85+	4212 (43.0)	0.88 (0.74, 1.05)	6248 (37.4)	1.05 (0.92, 1.19)
Marital Status				
Never married/widowed/separated/divorced/other	5798 (59.2)	1.69 (1.51, 1.88)	7999 (47.8)	1.78 (1.62, 1.95)
Married/civil union/defacto	3998 (40.8)	Ref	8721 (52.1)	Ref
Living arrangement ***				
Alone	624 (6.4)	Ref	5526 (33.0)	Ref
With spouse/partner only	888 (9.1)	1.91 (1.48, 2.47)	7419 (44.4)	2.15 (1.93, 2.40)
With spouse/partner and others	1521 (15.5)	1.29 (1.01, 1.65)	1500 (9.0)	1.73 (1.46, 2.05)
With child or others	6763 (69.0)	0.73 (0.58, 0.92)	2280 (13.6)	1.48 (1.27, 1.72)
Perceived to be better-off living elsewhere ***	6262 (63.9)	2.32 (2.04, 2.64)	4994 (29.9)	1.63 (1.48, 1.79)
Ethnicity				
Chinese	N/A	N/A	218 (1.3)	1.16 (0.80, 1.68)
Asian other than Chinese	N/A	N/A	212 (1.3)	0.86 (0.57, 1.31)
European	N/A	N/A	14,322 (85.6)	0.91 (0.80, 1.04)
Maori	N/A	N/A	1077 (6.4)	0.97 (0.81, 1.16)
Middle Eastern/Latin American/African	N/A	N/A	84 (0.5)	1.03 (0.56, 1.91)
Pacific peoples	N/A	N/A	687 (4.1)	0.71 (0.55, 0.91)
Others	N/A	N/A	118 (0.7)	1.34 (0.84, 2.16)
Language use				
Cantonese/ Mandarin/ Other Chinese dialect	9732 (99.3)	Ref	N/A	N/A
English	14 (0.1)	0.90 (0.20, 4.04)	N/A	N/A
Other dialect	50 (0.5)	0.60 (0.24, 1.52)	N/A	N/A
**Health and functional status of care-recipient**				
Stay in hospital within 7 days ***	1102 (11.2)	0.95 (0.83, 1.09)	4474 (26.8)	1.63 (1.48, 1.79)
Hearing*				
Adequate	3890 (39.7)	Ref	8815 (52.7)	Ref
Minimal difficulty	4229 (43.2)	0.83 (0.74, 0.94)	4499 (26.9)	1.00 (0.90, 1.11)
Moderate to severe difficulty	1677 (17.1)	1.09 (0.994, 1.27)	3407 (20.4)	1.35 (1.21, 1.51)
Vision ***				
Adequate	2932 (29.9)	Ref	12,155 (72.7)	Ref
Minimal difficulty	5743 (58.6)	0.99 (0.88, 1.12)	3261 (19.5)	1.19 (1.07, 1.33)
Moderate to severe difficulty	1121 (11.4)	0.75 (0.61, 0.91)	1305 (7.8)	1.41 (1.21, 1.64)
3+ IADL items with difficulties	9672 (98.7)	3.11 (1.56, 7.36)	15,901 (95.1)	3.29 (2.39, 4.53)
ADL Hierarchy Scale ***				
Independent	3723 (38.0)	Ref	7392 (44.2)	Ref
Supervision	2163 (22.1)	0.99 (0.85, 1.14)	4115 (24.6)	2.28 (2.03, 2.56)
Limited to total dependence	3910 (39.9)	0.87 (0.77, 0.99)	5217 (31.2)	2.63 (2.36, 2.93)
Fall in last 90 days	3029 (30.9)	1.17 (1.04, 1.31)	6356 (38.0)	1.38 (1.26, 1.50)
Behavioral problems in past 3 days ***	2317 (23.7)	1.90 (1.68, 2.13)	3162 (18.9)	2.61 (2.37, 2.87)
Mood problem in past 3 days ***	1304 (13.4)	1.74 (1.51, 2.00)	2117 (12.7)	2.51 (2.25, 2.80)
Comorbidity				
Dementia only	1715 (17.5)	Ref	10,008 (59.8)	Ref
Stroke & dementia	637 (6.5)	0.71 (0.54, 0.92)	1337 (8.0)	1.05 (0.89, 1.24)
CVD & dementia	4272 (43.6)	0.90 (0.78, 1.04)	4274 (25.6)	1.06 (0.95, 1.17)
CVD, stroke & dementia	3172 (32.4)	0.79 (0.68, 0.93)	1106 (6.6)	1.21 (1.02, 1.44)
**Caregiver characteristics**				
Primary caregiver relationship with care-recipient				
Child or child-in-law	5503 (56.2)	1.27 (1.08, 1.50)	7420 (44.4)	1.16 (0.97, 1.38)
Spouse/partner/significant other	2303 (23.5)	2.92 (2.46, 3.46)	7653 (45.8)	2.07 (1.73, 2.46)
Parent/guardian/sibling/other relative or whanau/friends	1990 (20.3)	Ref	1652 (9.9)	Ref
Primary caregiver lives with care-recipient ***	4629 (47.3)	1.50 (2.35, 1.68)	10,691 (63.9)	1.99 (1.79, 2.20)
Primary caregiver provides IADL care ***	9171 (93.6)	2.98 (2.17, 4.23)	14,535 (86.9)	0.80 (0.71, 0.91)
Primary caregiver provides ADL care	7440 (75.9)	3.22 (2.72, 3.85)	7979 (47.7)	1.64 (1.50, 1.79)
Primary caregiver provides more than 21 h of care in a week	2504 (25.6)	1.65 (1.47, 1.85)	7192 (43.0)	1.44 (1.32, 1.57)
**Formal care services utilization of care-recipient**				
Home services***	166 (1.7)	2.60 (1.86, 3.61)	4157 (24.9)	0.94 (0.85, 1.04)
Visiting nurse*	600 (6.1)	1.37 (1.11, 1.69)	963 (5.8)	0.98 (0.81, 1.18)
Allied-health services ***	407 (4.2)	0.45 (0.31, 0.64)	2074 (12.4)	1.50 (1.33, 1.69)
Hospital services **	5753 (58.7)	1.10 (0.98, 1.23)	5934 (35.5)	1.37 (1.25, 1.50)

*ADL* activities of daily living, *CVD* cardiovascular diseases, *IADL* instrumental activities of daily living

*P*-value of interaction value is denoted as *: < 0.05; **:< 0.01; ***:< 0.001

### Demographic information of care-recipient

The demographic characteristics of the two regions are shown in Table 1. In both regions, slightly more females (HK: 60.2%; NZ: 57.2%) and care-recipients aged 75–84 years old (HK: 45.3%; NZ: 46.1%) were presented. In HK, majority of care-recipient lived with child (69.0%) whereas majority lived with spouse/partner (44.4%) in NZ. Over 80% of the care-recipients in our NZ sample was European, comparing to a majority of Chinese speakers in the Hong Kong sample (99.3%). In a stratified analysis, we also found that the demographic characteristics of Chinese samples in the NZ, and that of the HK population shared a lot of similarities (as illustrated in S3).

### Health and functional status of care-recipient

Higher proportion of care-recipient had poor sensory function including hearing and vision in HK (60.3, 70.0% respectively) than NZ (47.3, 27.3% respectively). Care-recipients in HK had higher proportion of behavioural problem (HK: 23.7%, NZ: 18.9%), ADL dependency (HK: 62%; NZ: 55.8%) and mood problem (HK: 13.4%; NZ: 12.7%) when comparing to NZ. In both regions, there was very high proportion of care-recipient with three or more IADL difficulty observed (98.7% in HK, 95.1% in NZ). About one-third of care-recipient had experience of fall in the past 90 days in both regions (30.9% in HK, 38.0% in NZ). There were more care-recipients co-morbid with cardiovascular disease or stroke in HK (82.5%) while comparing with NZ (40.2%).

### Caregiver characteristics

More than half of the care-recipients was cared by their immediate family such as child, child-in-law, spouse, partner, or significant others (HK: 79.7%; NZ: 90.1%). More primary caregivers lived with their care-recipients in NZ (63.9%) when comparing to HK (47.3%). Most of the primary caregivers provided IADL care in both region (HK: 93.6%; NZ: 86.9%), and higher proportion of caregiver provided ADL care in HK (75.9%) than NZ (47.7%).

### Formal care services utilization of care-recipient

Care-recipients in both regions used more medical care (98.3% in HK, 75.1% in NZ) than social care. Both care-recipients in HK and NZ relied mainly on hospital service (HK: 58.7%; NZ: 35.5%). Comparing to NZ, HK used less visiting nurse (HK:12.5%; NZ: 5.8%) and allied-health services (HK:6.5%; NZ:12.4%). Formal care service utilization pattern of different ethics/language user groups in HK and NZ is illustrated in S4.

### Factors contributing to caregiver burnout

Table 2 shows the results of logistic regression model on caregiver burnout. Several significant factors were found to be contributing to caregiver burnout in either one of the regions. Care-recipients’ ADL dependency and fall history in 90 days, and primary caregiver living with care-recipients were significantly associated with caregiver burnout in the NZ population only. In the HK population, primary caregiver being child or child-in-law, and the utilization of home care services and hospital service were significantly associated with additional risk of caregiver burnout. In terms of the effect size, the factor primary caregiver “being spouse/partner/significant other of the care-recipient” had the largest AOR in the HK model (AOR = 3.18); while in the NZ model, the factor “care-recipient perceived to be better-off living elsewhere” had the largest AOR (AOR = 5.68).

**Table 2:** Results of the force-entry logistic regression model with all covariates

	HK	NZ
	AOR (95CI)	AOR (95CI)
**Demographic Information of care-recipient**		
Female	1.00 (0.87, 1.15)	0.86 (0.78, 0.96)**
Age		
65–74	Ref	Ref
75–84	1.08 (0.89, 1.31)	0.99 (0.86, 1.14)
85+	1.21 (0.99, 1.48)	0.97 (0.84, 1.13)
Marital Status		
Never married/Widowed/Separated/Divorced/Other	0.89 (0.73, 1.09)	0.95 (0.76, 1.19)
Married/Civil Union/Defacto	Ref	Ref
Living arrangement		
Alone	Ref***	Ref
With spouse/partner only	0.88 (0.61, 1.27)	1.40 (1.08, 1.83)*
With spouse/partner and others	0.74 (0.52, 1.06)	1.27 (0.97, 1.66)
With child	0.61 (0.47, 0.79)***	1.02 (0.77, 1.35)
Perceived to be better-off living elsewhere	2.76 (2.40, 3.17)***	5.68 (5.09, 6.34)***
**Health and functional status of care-recipient**		
Stay in hospital within 7 days	0.95 (0.78, 1.15)	0.88 (0.75, 1.03)
Hearing		
Adequate	Ref***	Ref*
Minimal difficulty	0.82 (0.71, 0.93)**	0.92 (0.82, 1.04)
Moderate to severe difficulty	1.16 (0.98, 1.37)	1.10 (0.97, 1.25)
Vision		
Adequate	Ref***	Ref
Minimal difficulty	0.99 (0.87, 1.14)	0.99 (0.87, 1.11)
Moderate to severe difficulty	0.67 (0.53, 0.84)***	1.01 (0.85, 1.20)
3+ IADL items with difficulties	2.00 (0.90, 4.43)	1.15 (0.82, 1.62)
ADL Hierarchy Scale		
Independent	Ref	Ref***
Supervision	0.98 (0.84, 1.15)	1.43 (1.26, 1.63)***
Limited to Total dependence	1.03 (0.88, 1.21)	1.31 (1.14, 1.49)***
Fall in last 90 days	1.01 (0.89, 1.15)	1.53 (1.37, 1.70)***
Behavioral problems in past 3 days	1.61 (1.41, 1.83)***	1.75 (1.55, 1.98)***
Mood problem in past 3 days	1.53 (1.30, 1.79)***	1.09 (0.99, 1.21)
Comorbidity		
Dementia only	Ref***	Ref
Stroke & Dementia	0.67 (0.51, 0.89)**	0.88 (0.74, 1.05)
CVD & Dementia	0.93 (0.79, 1.09)	1.02 (0.91, 1.15)
CVD, Stroke & Dementia	0.75 (0.63, 0.89)**	0.94 (0.78, 1.13)
**Caregiver characteristics**		
Primary caregiver relationship with care-recipient		
Child or child-in-law	1.55 (1.29, 1.86)***	1.19 (0.97, 1.47)
Spouse/Partner/significant other	3.18 (2.52, 4.00)***	1.49 (1.12, 2.00)**
Parent/guardian/Sibling/Other relative or whanau/friends	Ref***	Ref*
Primary caregiver lives with care-recipient	0.93 (0.78, 1.11)	1.69 (1.32, 2.15)***
Primary caregiver provides IADL care	1.22 (0.84, 1.77)	0.82 (0.71, 0.96)*
Primary caregiver provides ADL care	2.92 (2.39, 3.56)***	1.20 (1.06, 1.36)**
Primary caregiver provides more than 21 h of care in 7 days	1.57 (1.35, 1.81)***	1.35 (1.20, 1.52)***
**Formal care services utilization of care-recipient**		
Home care service (home making or meal services)	1.84 (1.27, 2.65)**	1.06 (0.86, 1.30)
Visiting Nurse	1.39 (1.10, 1.74)**	1.22 (1.09, 1.38)***
Allied-health Service	0.49 (0.34, 0.72)***	0.98 (0.84, 1.14)
Hospital Service (inpatient care or emergency unit visit)	1.17 (1.02, 1.33)*	1.00 (0.88, 1.14)

*ADL* activities of daily living, *IADL* instrumental activities of daily living

*P*-values is denoted as *: < 0.05; **:< 0.01; ***:< 0.001

Perception of care-recipient would be better-off living elsewhere, care-recipient having hearing problem and behavioural problem in the past 3 days, primary caregiver being a spouse/partner/significant other, primary caregiver provides ADL care, primary caregivers providing more than 21 h of care every week, and care-recipients using visiting nurse services had significant contributions to caregiver burnout in both HK and NZ.

Allied health service utilization was found to be protective to caregiver burnout in HK (AOR = 0.49, *p*-value < 0.001) and in NZ (AOR = 0.98). Utilization of other formal services, including home care (AOR in HK = 1.84; AOR in NZ = 1.06), visiting nurse (AOR in HK = 1.39; AOR in NZ = 1.22) and hospital services (AOR in HK = 1.17; AOR in NZ = 1.00) were associated with caregiver burnout.

### Moderation between allied-health services and other co-variates

**Fig. 1:**
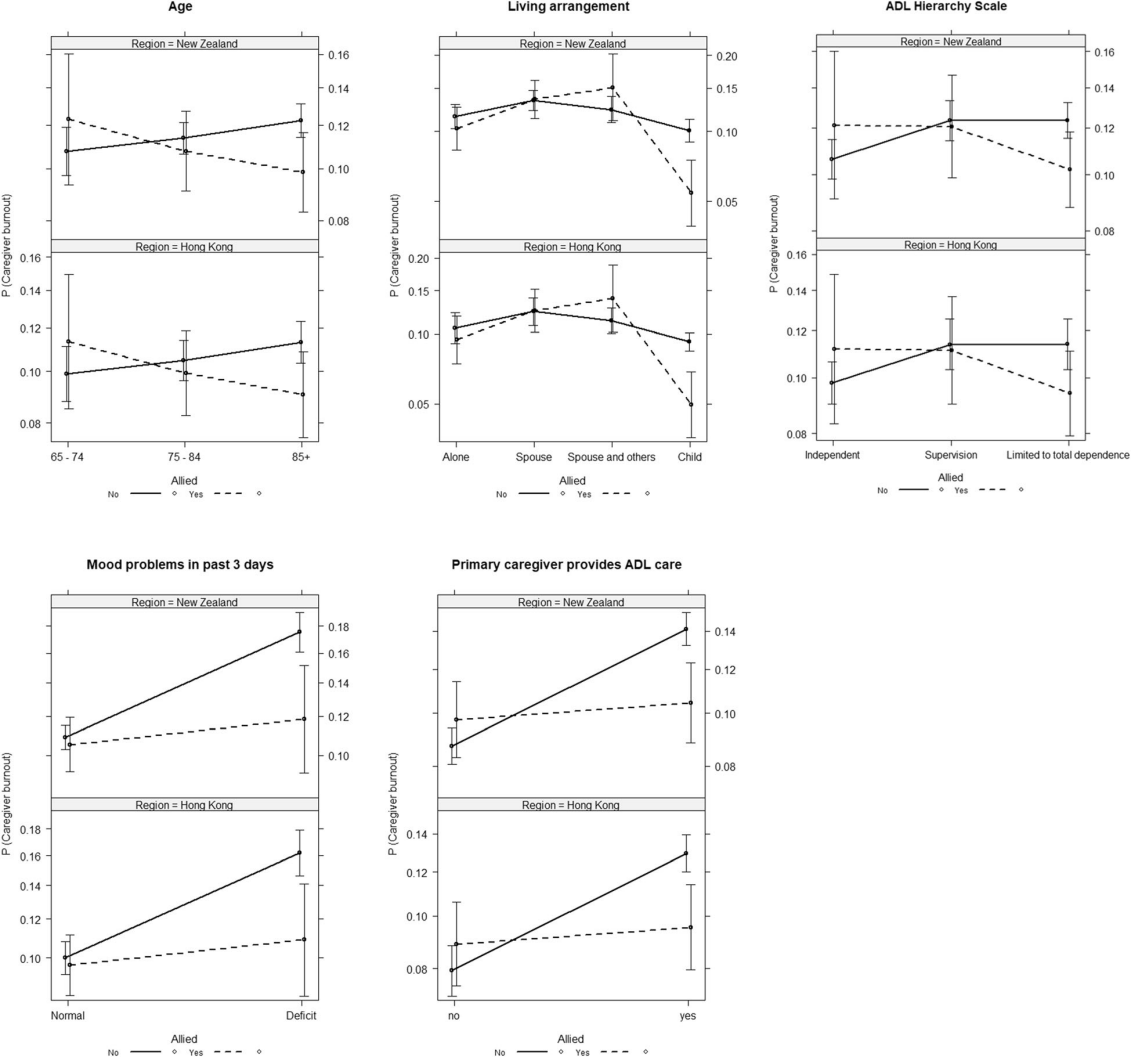
Interactions between allied-health utilization and the factors associated with caregiver burnout

Table 3 shows the results of the stratified analysis between caregivers of care-recipient who had used or not used allied-health services. Figure 1 shows the moderation analysis between allied-health services utilization and the factors contributing to caregiver burnout. The following interaction terms between allied-health services and potential contributing factors were found to be significant after adjusted for other co-variates: care-recipient’s age (AOR = 0.67, 95% CI = 0.47–0.97, *p*-value< 0.05), living arrangement (AOR = 0.58, 95% CI = 0.38–0.87, *p*-value< 0.05), ADL dependency (AOR = 0.69, 95% CI = 0.49–1.00, *p*-value< 0.05), mood problem in the past 3 days (AOR = 0.66, 95% CI = 0.48–0.90, p-value< 0.01), and primary caregiver providing ADL care (AOR = 0.63, 95% CI = 0.49–0.80, p-value< 0.001). Sensitivity analysis using common significant factors in both NZ and HK was illustrated in S5 and S6.

**Table 3:** Results of multi-group analysis

	Not using allied health services		Using allied health services	
	HK	NZ	HK	NZ
	AOR (95CI)	AOR (95CI)	AOR (95CI)	AOR (95CI)
**Demographic Information of care-recipient**				
Female	1.01 (0.87, 1.16)	0.87 (0.78, 0.98)*	0.46 (0.16, 1.34)	0.90 (0.70, 1.17)
Age				
65–74	Ref	Ref	Ref	Ref
75–84	1.10 (0.90, 1.33)	1.02 (0.88, 1.18)	0.63 (0.17, 2.32)	0.88 (0.61, 1.28)
85+	1.23 (1.00, 1.51)*	1.04 (0.88, 1.22)	0.51 (0.12, 2.22)	0.83 (0.57, 1.23)
Marital Status				
Never married/Widowed/Separated/Divorced/Other	0.89 (0.72, 1.09)	1.05 (0.82, 1.33)	1.23 (0.25, 6.07)	0.59 (0.32, 1.09)
Married/Civil Union/Defacto	Ref	Ref	Ref	Ref
Living arrangement				
Alone	Ref***	Ref	Ref	Ref
With spouse/partner only	0.88 (0.61, 1.28)	1.41 (1.06, 1.88)*	0.10 (0.01, 1.73)	1.19 (0.57, 2.49)
With spouse/partner and others	0.73 (0.51, 1.04)	1.16 (0.87, 1.55)	0.13 (0.01, 1.93)	1.60 (0.78, 3.28)
With child	0.60 (0.47, 0.78)***	1.06 (0.78, 1.43)	0.07 (0.01, 0.54)*	0.56 (0.27, 1.19)
Perceived to be better-off living elsewhere	2.74 (2.38, 3.15)***	5.83 (5.19, 6.56)***	3.90 (1.36, 11.15)*	4.68 (3.41, 6.42)***
**Health and functional status of care-recipient**				
Stay in hospital within 7 days	1.05 (0.87, 1.28)	0.84 (0.73, 0.97)*	0.06 (0.01, 0.45)**	0.86 (0.56, 1.35)
Hearing				
Adequate	Ref***	Ref*	Ref	Ref
Minimal difficulty	0.81 (0.70, 0.92)**	0.93 (0.82, 1.06)	0.87 (0.31, 2.46)	0.94 (0.70, 1.26)
Moderate to severe difficulty	1.15 (0.97, 1.37)	1.15 (1.00, 1.33)*	1.22 (0.29, 5.22)	0.99 (0.73, 1.36)
Vision				
Adequate	Ref***	Ref	Ref	Ref
Minimal difficulty	1.01 (0.88, 1.16)	1.01 (0.88, 1.15)	0.39 (0.14, 1.09)	0.94 (0.71, 1.26)
Moderate to severe difficulty	0.67 (0.53, 0.85)***	1.11 (0.92, 1.33)	0.96 (0.25, 3.73)	0.69 (0.46, 1.05)
3+ IADL items with difficulties	1.98 (0.89, 4.39)	1.20 (0.84, 1.71)	NA	0.59 (0.12, 2.89)
ADL Hierarchy Scale				
Independent	Ref	Ref***	Ref	Ref
Supervision	1.01 (0.86, 1.18)	1.44 (1.25, 1.65)***	1.14 (0.26, 4.97)	1.10 (0.71, 1.69)
Limited to Total dependence	1.09 (0.93, 1.27)	1.35 (1.17, 1.56)***	1.86 (0.49, 7.09)	0.91 (0.59, 1.39)
Fall in last 90 days	1.05 (0.92, 1.19)	1.11 (0.99, 1.24)	1.47 (0.57, 3.75)	1.05 (0.82, 1.36)
Behavioral problems in past 3 days	1.59 (1.40, 1.81)***	1.56 (1.38, 1.76)***	2.33 (0.75, 7.23)	1.35 (1.04, 1.77)*
Mood problem in past 3 days	1.53 (1.31, 1.80)***	1.89 (1.65, 2.15)***	1.64 (0.43, 6.28)	1.18 (0.85, 1.64)
Comorbidity				
Dementia only	Ref***	Ref	Ref	Ref
Stroke & Dementia	0.70 (0.53, 0.93)*	0.83 (0.68, 1.02)	0.30 (0.03, 3.66)	1.00 (0.69, 1.45)
CVD & Dementia	0.94 (0.80, 1.11)	1.05 (0.93, 1.18)	0.74 (0.21, 2.66)	0.93 (0.70, 1.25)
CVD, Stroke & Dementia	0.76 (0.64, 0.91)**	0.92 (0.74, 1.13)	0.64 (0.17, 2.39)	1.10 (0.73, 1.68)
**Caregiver characteristics**				
Primary caregiver relationship with care-recipient				
Child or child-in-law	1.58 (1.32, 1.90)***	1.15 (0.91, 1.44)	0.64 (0.17, 2.36)	1.34 (0.84, 2.14)
Spouse/Partner/significant other	3.22 (2.54, 4.07)***	1.32 (0.96, 1.81)	2.55 (0.61, 10.62)	2.57 (1.22, 5.41)*
Parent/guardian/Sibling/Other relative or whanau/friends	Ref***	Ref	Ref	Ref*
Primary caregiver lives with care-recipient	0.92 (0.77, 1.10)	1.57 (1.21, 2.05)***	0.87 (0.19, 3.95)	2.33 (1.24, 4.38)**
Primary caregiver provides IADL care	1.19 (0.82, 1.74)	0.83 (0.69, 1.00)*	3.82 (0.26, 57.02)	0.84 (0.62, 1.13)
Primary caregiver provides ADL care	3.02 (2.47, 3.69)***	1.24 (1.08, 1.41)**	0.57 (0.12, 2.80)	1.00 (0.73, 1.38)
Primary caregiver provides more than 21 h of care in 7 days	1.52 (1.31, 1.77)***	1.34 (1.18, 1.52)***	2.46 (0.80, 7.63)	1.22 (0.85, 1.75)

*ADL* activities of daily living, *IADL* instrumental activities of daily living

*P*-value of interaction value is denoted as *: < 0.05; **:< 0.01; ***:< 0.001

## Discussion

### Implications of service delivery models on dementia caregiving experience in HK and NZ

Our study included all dementia caregivers whose care-recipient were applying for social care support in HK and NZ, and therefore could fill the knowledge gap by providing an understanding of dementia caregiver burnout in two ethnically and contextually distinct regions. The prevalence of caregiver burnout is found to be 15.5 and 13.9% in HK and NZ respectively. It is an important issue in connection with ageing, which may be accountable to the difference in service delivery of the two regions as the followings.

Despite the similar proportion of government expenditure on healthcare, caregiver support services were delivered differently in NZ and HK, which could contribute to the difference in the dementia caregiving experience in the two regions. In 2014/15, 5.95% of the Gross Domestic Product of NZ government was spent on health [30], while the health expenditure in HK accounts for 5.9% of the Gross Domestic Product in 2016/17 [42]. In NZ, home based support services were delivered by DHB contracted agencies after they have assessed the care needs of both the care-recipient and caregiver; and the care plan and assessment is reviewed annually to ensure an appropriate level of support is allocated. Health services for dementia care in NZ are guided by the New Zealand Framework for Dementia Care and are primarily funded by the central government based on a community-oriented mode, with 20 district health boards (DHBs) of varying sizes being responsible for funding and/or providing health services in their district to meet service needs including primary and secondary care services. In HK, support services are delivered without a standardized needs assessment nor dementia care framework for the caregivers, and the provision of community services is scarce. There is a lack of comprehensive framework to guide service planning and provide coordination among those centres as well as communication with the Department of Health. This resulted in issues such as accessibility related to long waiting time, service gap to fill in social need, limited capacity quota and lack of sustainability [43]. Study found that caregivers tend to prioritize the benefits of their loved ones over their own health [8], thus long waiting time and inadequate community service in HK may further isolate caregivers from formal support and contribute to additional burden. Therefore, it is critical to identify those in need and provide efficient and appropriate service support for caregivers. The lack of standardized and routinely conducted need assessment of caregiver burden in HK social system could lead to delayed access to formal support services, which possibly contributes to the higher prevalence of dementia caregiver burnout in HK. A comprehensive needs assessment for caregivers, and expanding inter-disciplinary care provided by geriatrician and allied-health professionals might help support caregivers better.

### Differences in contributing factors in HK and NZ

Our study found that care-recipients’ perception to be better-off living elsewhere contribute to higher risk to caregiver burnout in NZ, which could be explained by the different family structures and ethnical compositions in the two regions. There were more people living alone in the NZ sample than that in HK, which suggests cohabitation maybe unusual in NZ and could bring additional stress to caregivers. This observation is supported by other studies in Western countries where cohabitation was found to be a risk factor to caregiver stress in caregivers of people living with dementia [44]. Living in the same environment with the care-recipients could bring increased chance for conflicts and even abusive behaviours to the caregiving dyad, and therefore may give more emotional burden to the caregivers of people with dementia [45]. Chinese caregivers might be more resilient to the stress brought by co-residence with the care-recipients comparing to the Europeans, since Chinese family members are traditionally living together and are culturally-bounded to fulfil their care responsibility to the senior members. Living with their care-recipients may also be deemed as a fulfilment of filial responsibility of the caregiver, which could explain the insignificant protective role of cohabitation in the HK model (AOR = 0.93).

Another difference identified was that the ADL dependence of care-recipient had a significant association with caregiver burnout in NZ sample only, despite the fact that there are higher proportions of ADL-dependent care-recipients and more primary caregiving providing ADL care in HK. This could be explained by the difference in cultural values in the two populations. The concept of filial piety and the caregiving responsibility deeply rooted in the culture of the Chinese may be responsible for the high percentage of ADL care provision in HK. The collectivist values embedded in Confucianism impose the caregiving duty of older people on their family members and to encourage individual to sacrifice personal priorities for the betterment of the seniors in their family [46]. Chinese primary caregivers are engaged in personal care, including ADL caregiving items (e.g. washing, toileting, meals), in the hope of better take care of their seniors despite the repulsiveness of the tasks [17], which might result in better tolerance to ADL caregiving tasks while comparing to their NZ counterparts.

Our cross-region comparison of caregiver burnout in HK and NZ highlights that although dementia is a neurodegenerative conditions affecting people of all ethnic groups, different sociocultural factors (including stigma) are likely to be operating and impact on the presentation of dementia and healthcare utilization in different ethnic groups [47]. Understanding and addressing these unique socio-cultural issues is therefore an important part of the practice of person-centred care and promoting the concept of living well with dementia. Further qualitative research is needed to understand the relationship between aged residential care placement decision and caregiver burnout with the ethical and moral perspectives on the family structure of living with people with dementia in NZ and HK. *Similarities in contributing factors in HK and NZ.*

Besides its contributions to understand the cross-regional differences in dementia caregiving experience through service delivery models, family structures and cultural values, this study found some common factors contributing to the caregiver burnout in both HK and NZ. In line with the findings of previous literature, behavioural symptoms of the care-recipients contribute to the caregiver burnout in both region s[1].. Behavioural symptoms of care-recipients is a determinant of the caregiver-care recipient relationship closeness [48], where a high relationship closeness was found to be protective to caregivers’ physical and mental health outcomes [49]. Occupational-therapist-led intervention was found to have significant effect on reducing caregiver burden by better managing the mood and behavioural symptoms of the care-recipients [28] Behavioural symptoms management could be strengthened in the provision of dementia caregiver support services to reduce burnout.

The perception of care-recipients would be better-off living elsewhere was found to be a significant proxy of caregiver burnout in both regions, with a larger effect size in the NZ sample. Evidence suggested the decision of nursing home placement is link with caregiver burden, which aroused from guilt and regret of the institutionalization decision [50]. Caregivers could also be vulnerable during the decision making process due to the limited support, little information about alternate care and financial options, and not informed about the knowledge of dementia [51]. These additional stresses could add to the risk of caregiver burnout associated with the perception of care-recipient would be better-off living in elsewhere. Person and caregiver-centric principles could be introduced in the planning and provision of dementia care in order to respond to needs of the caregiving dyads and allow autonomy in making decisions in care plan.

We found that dementia caregivers who had longer caregiving time were more vulnerable to develop burnout in both regions. This is consistent with previous study that caregivers who provide intense care are at higher risk of caregiver burnout due to the intensity of the caregiving task s[52]. A long caregiving time may also mean that the caregivers have to give up more personal time and working time for taking care of the care-recipients. The provision of an efficient caregiver support service is therefore vital to maintain the quality-of-life of the caregiving dyads, by assisting the caregivers to provide care and allowing them to get respite from the long caregiving time. A systematic review concluded that multi-component interventions could significantly reduce caregiver burden despite the physically-demanding caregiving tasks; while symptoms management advice given by nursing and health professionals were found to be effective in relieving burden in dementia caregivers [53]. In NZ, caregiver support interventions including financial assistance, transportation arrangement, respite care subsidy and community services are funded by the Ministry of Health and Ministry of Social Development. In HK, caregiver support services are delivered by the community services including caregiver training and respite care support under Social Welfare Department. Incorporating ADL care relevant elements into the dementia caregiver support services would be an important strategy to reduce caregiver burnout.

Primary caregiver being spouse/partner/significant others was found to be associated with caregiver burnout in HK and NZ. Spousal caregivers have higher chance of depression and are more vulnerable to caregiver burnout comparing to other caregivers [44]. Scholar suggested the social isolation due to caregiving, the continuous care burden, and the progressive loss of their partner may be the reasons of spousal caregivers’ vulnerability [54]. Spousal caregivers are usually elder than other caregivers (e.g. offspring and friends), where their elder age may pose additional challenges to the caregiving work comparing to their younger counterparts. There is evidence suggesting public services utilization, including allied health support, could be beneficial to reducing caregiving burden [55].

### Protective effect of allied health service utilization

Our study found a unique protective effect of allied-health support services for dementia caregiver burnout in HK. Allied-health service utilization was found to be a moderator between caregiver burnout and a series of contributing factors, including care-recipient’s age, living arrangement, ADL Hierarchy scale, mood problem, and primary caregivers’ ADL care provision. Allied-health services such as physiotherapy and occupational therapy were found to significantly lower caregiver burden [28], slow down ADL decline in older adults [28], and can sometimes improve their functional outcomes in spite of their limited ability to learn [56, 57]. Other studies have suggested that functional deficits decrease the quality-of-life in care-recipients with dementia [58]; while information support provided by the allied-health could improve the quality-of-life of care-recipients and their caregivers [57], and help caregivers to acquire knowledge gain mastery over their care duties [59]. These findings support the provision of allied-health services to maintain the quality-of-life of community-dwelling adults with dementia and their caregivers. Based on our findings, allied-health support for people with dementia and their caregivers could be a strategy to address caregiver burnout in HK.

### Limitations

This study has several limitations that need to be acknowledged. Firstly, demographic information of caregivers, for example marital status and employment, was not routinely collected by the interRAI assessment in HK and NZ, which limited the ability to explain caregiver burnout in this model. Secondly, the InterRAI assessment only included older adult who was seeking public-funded services in HK and NZ, therefore this study may limit the analysis about care-recipient with other age or those did not seek public support. However, this study provided important knowledge about those who in desperate need of support. Thirdly, the cross-sectional design of this study could not provide causality link between contributing factors and caregiver burnout. Also, the difference between the AOR of the perception that the person living with dementia would be better of living elsewhere in the two regions, would require more qualitative investigation to provide explanation. Lastly, caregiver burnout in this study was a binary outcome and a continuous measure of burnout severity is not part of the interRAI assessment. Caregiver burnout is a result of accumulating burden during the caregiving process [1], and such progressive nature of burnout may not be accurately captured by a binary outcome. Binary outcomes also have different statistical considerations in comparing to continuous or ordinal outcomes, such as applying a logit link function in the regression, and a lower statistical power. In this secondary data analysis, the reporting of the binary outcome is confined by the data type collected in the pre-defined assessment tool. In order to remedy the shortcomings in reporting binary outcomes, Rombach and colleagues [60] recommended medical researchers to use statistical methods that could quantify the confidence intervals of the primary binary outcomes whenever available (including logistic regression in our study), instead of choosing analysis method that could only produce a corresponding *p*-value (such as chi-square test).

To the best of our knowledge, this is the first study that provided evidence on the differences (in term of social system, family structure, concept of filial piety) and similarities (in term of characteristics of caregiving and important role of continuity healthcare professional service on contributing factors of caregiver burnout related to dementia) in two different regions that include all caregiver whose care-recipient applied for publicly-funded support services. The structured needs assessment of caregiver in NZ and allied-health services in HK are highlighted as protective factor on caregiver burnout. In response to the limitation of using binary outcome in this study, future research can be conducted to investigate the longitudinal impact of allied-health services on caregiver burnout along the trajectory of dementia in HK and NZ by using a validated continuous or ordinal measure (such as the Zarit Burden Interview [61, 62]). Under the circumstances of limited data availability in secondary data analysis, researchers should adopt a statistical analysis method that could produce a confidence interval of the binary outcomes whenever available. Also, qualitative investigation could be used to understand the facilitators and barriers of using allied-health services in HK and NZ in the hope to guide the development of context- and needs-specific dementia care policies.

## Conclusion

This research provided a cross-region comparison on the contributing factors of caregiver burnout in dementia using territory-wide data in HK and NZ. It hints the unique protective effect of allied-health services in Hong Kong, described the characteristics of care-recipients whose caregiver may be at risk of burnout, and accounted for the different burden-contributing factors in their context-specific content. Our findings provide important input on the reference of dementia care to develop context-specific care strategy, and allow geriatricians to identify the caregivers who may be at risk of burnout for providing early intervention to older adults with dementia and their caregivers.

## Supplementary Information

**Additional file 1: Supporting Information 1.** A brief history of the interRAI and quality assurance mechanisms in Hong Kong and New Zealand. **Supporting Information 2.** Regrouping of variables**. Supporting Information 3.** Demographic information of community-dwelling elderly aged 65+ who was diagnosed with dementia. **Supporting Information 4.** Service utilization by ethnics or primary language use. **Supporting Information 5.** Results of the logistic regression model including factors that were significant in both HK and NZ. **Supporting Information 6.** Results of multigroup analysis with contributing factors that were significant in both HK and NZ.

## Data Availability

The datasets generated and analysed during the current study are not publicly available due them containing information that could compromise research participant privacy/consent, but are available from the Social Welfare Department of Hong Kong and the Central Region’s Technical Advisory Services of New Zealand on reasonable request.

## Abbreviations

CI: Confidence interval
CVD: Cardiovascular disease
DHBs: District health boards
HK: Hong Kong
InterRAI: International Residential Assessment
interRAI-HC 9.1: interRAI Home Care Assessment version 9.1
interRAI MDS-HC: interRAI Minimal Data Set – Home Care Assessment Version 2.0
NZ: New Zealand
AOR: Adjusted odds ratio
SPSS: IBM Statistical Package for the Social Sciences
SWD: Social Welfare Department of Hong Kong
