# Supplementary material for: Caregiving burnout of community-dwelling people with dementia in Hong Kong and New Zealand: a cross-sectional study
Source: BMC Geriatr. 2021 Apr 20;21:261. doi: 10.1186/s12877-021-02153-6 (PMC8059033; doi:10.1186/s12877-021-02153-6)
Supplement: Supplementary file 1 — Additional file 1: Supporting Information 1. A brief history of the interRAI and quality assurance mechanisms in Hong Kong and New Zealand. Supporting Information 2. Regrouping of variables. Supporting Information 3. Demographic information of community-dwelling elderly aged 65+ who was diagnosed with dementia. Supporting Information 4. Service utilization by ethnics or primary language use. Supporting Information 5. Results of the logistic regression model including factors that were significant in both HK and NZ. Supporting Information 6. Results of multigroup analysis with contributing factors that were significant in both HK and NZ. [file 12877_2021_2153_MOESM1_ESM.docx]

Supporting Information 1. A brief history of the interRAI and quality assurance mechanisms in Hong Kong and New Zealand.

Designed to assess the functional status and quality of life issues of community-dwelling adults, the interRAI Home Care (HC) can be used to inform and guide service planning. [1] interRAI HC has demonstrated good inter-rater reliability in several countries, and it is currently being used in North America (Canada and multiple states in the U.S.), Europe (Italy, Switzerland, Finland, Estonia, etc.), and Asia/Pacific Rim (Hong Kong, Singapore, Japan, Australia, New Zealand).

Hong Kong, China

In Hong Kong, information of the older adults (age≥65 years old) and their caregivers were assessed with the interRAI Minimal Data Set – Home Care Assessment Version 2.0 (MDS-HC) as a part of “the Standardised Care Need Assessment Mechanism for Elderly”. interRAI MDS-HC was specifically developed for the home care setting.[1] The Assessment was performed by accredited assessors from difference disciplines such as social workers, nurses, occupational therapists and physiotherapists.[2] The interviewers have received training and accreditation before they conduct the assessment to ensure the inter-rater reliability.[3] Since 2003, Hong Kong Social Welfare Department adopted MDS-HC to assess frail older adults who are seeking formal healthcare and supportive services to ensure better allocation of resources.[3]

New Zealand

The New Zealand study sample consisted of older people (age≥65 years old) who had received an interRAI Home Care Assessment version 9.1 (interRAI-HC 9.1). The New Zealand interRAI group at the Ministry of Health provided access to de-identified data from people who gave consent to have their records used for research purposes at the time of their interRAI assessment. A previous study using the New Zealand interRAI database has found 93.1% of people provided consent for research.[4] From 2012 New Zealand implemented interRAI for all older adults who are being considered for access to publically-funded community services or residential care. The New Zealand interRAI research database is large and ethnically diverse. [4] The interRAI data were collected by trained interRAI assessors using a face-to-face assessment. The interRAI assessor may use multiple sources of information e.g. referral note, person interview, observation, discussion with family, caregivers, or health professionals to gain accurate information. A national competency framework provides quality assurance for interRAI assessment. The interRAI assessors must be clinically registered and signed off as competent involving attending a 3-day interRAI training program, completing ten assessments and care plans, passing an evaluation, and achieving an acceptable quality review outcome.

Reference:

1. Morris JN, Fries BE, Bernabei R, Steel K, Ikegami N, Carpenter I. RAI-Home Care (RAI-HC) assessment manual for version 2.0. Marblehead, MA: Opus Communications; 2000.

2. Social Welfare Department Hong Kong. Standardised care need assessment mechanism for elderly services [Internet]. 2005. Available: https://www.swd.gov.hk/en/index/site_pubsvc/page_elderly/sub_standardis/

3. Social Welfare Department Hong Kong. Review of Standardised Care Needs Assessment Mechanism for Elderly Services. 2014.

4. Schluter PJ, Ahuriri-Driscoll A, Anderson TJ, Beere P, Brown J, Dalrymple-Alford J, et al. Comprehensive clinical assessment of home-based older persons within New Zealand: an epidemiological profile of a national cross-section. Aust N Z J Public Health. 2016;40: 349–355. doi:10.1111/1753-6405.12525

Supporting Information 2. Regrouping of variable

| Variables | Original categories | Regrouped categories |
| --- | --- | --- |
| Sex | 1. Female 2. Male | N/A |
| Age | Numeric entry | 1. 65-74 2. 75-84 3. 85+ |
| Marital status | 1. Never married 2. Married 3. Widowed 4. Separated 5. Divorced 6. Other 7. Civil Union† 8. Defacto † | 1. Never married/Widowed/Separated/Divorced/Other 2. Married/Civil Union/Defacto |
| Whom the care recipient lives with | 1. Alone 2. With spouse/partner only 3. With spouse/partner and others 4. With child | N/A |
| Whether the care-recipient is perceived to be better-off living elsewhere | 1. No 2. Only the caregiver 3. Only the care-recipient 4. Both the caregiver and the care-recipient | 1. Yes 2. No |
| Whether the care-recipient stay in hospital in last 7 days | The original variable was titled ‘Time since discharge from last in-patient setting’ with the following responses:   1. No hospitalization within 180days 2. Within last week 3. Within 8 to 14 days 4. Within 15 to 30 days 5. More than 30 days ago 6. Now in hospital† | 1. Yes 2. No |
| Hearing ability | 1. Adequate 2. Minimal difficulty 3. Moderate difficulty 4. Severe difficulty 5. No hearing † | 1. Adequate 2. Minimal difficulty 3. Moderate to severe difficulty |
| Vision ability | 1. Adequate 2. Minimal difficulty 3. Moderate difficulty 4. Severe difficulty 5. No vision † | 1. Adequate 2. Minimal difficulty 3. Moderate to severe difficulty |
| 3+ IADL items with difficulties | The 7 original IADL items had the following responses:   1. Independent 2. Some help 3. Full help 4. By others 5. Activity did not occur   Items that evaluated as not independent were recoded as ‘with difficulties’. The new variable was computed by counting whether the care-recipients have three or more IADL items with difficulties. | 1. Yes 2. No |
| ADL Hierarchy Scale | 1. Independent 2. Supervision 3. Limited 4. Extensive 5. Maximal 6. Dependent 7. Total Dependence | 1. Independent 2. Supervision 3. Limited to total dependence |
| Falls in last 90 days | Numeric entry | 1. Yes 2. No |
| Mood problem | HK/ NZ  HK E1a = NZ E1b  HK E1b = | 0 Not exhibit in last 3 days  1 Exhibited 1-2 of last 3 days  2 Exhibited on each of last 3 days |
| Behavioural problem |  |  |
| Comorbidity | The original variable was ‘whether the care-recipient was diagnosed with the following diseases: Alzheimer’s, Dementia other an Alzheimer’s diseases, Stroke, congestive heart failure, coronary artery diseases, and irregularly irregular pulse, peripheral vascular diseases.   1. Not present 2. Present: not subject to focused treatment or monitoring by home care professional 3. Present: monitored or treated by home care professional | 1. Dementia only 2. Stroke & Dementia 3. Cardiovascular disease (CVD) & Dementia 4. CVD, Stroke & Dementia |
| Primary caregiver relationship with care-recipient | 1. Child or child-in law 2. Spouse 3. Partner/significant other† 4. Parent/guardian† 5. Other relative*/Other relative or whanau† 6. Sibling† 7. Friend 8. Neighbour 9. Others | 1. Child or child-in-law 2. Spouse/Partner/significant other 3. Parent/guardian/Sibling/Other relative or whanau/friends |
| Primary caregiver lives with care-recipient | 1. No 2. Yes* 3. Yes, 6months or less† 4. Yes, more than 6 months† | 1. Yes 2. No |
| Primary caregiver provides IADL care | 1. Yes 2. No | N/A |
| Primary caregiver provides ADL care | 1. Yes 2. No | N/A |
| Primary caregiver provides more than 21hours of care in 7days | The original variable was ‘Number of care provided by the caregivers’ with a numeric response | 1. Yes 2. No |
| Home services  (Home-making or meals services) | The original variable was ‘Number of care provided to the care-recipient’ with a numeric response | 1. Yes 2. No |
| Visiting nurse |  |  |
| Allied-health services  (Physiotherapy, speech therapy or occupational therapy) |  |  |
| Hospital services  (Hospital admission or emergency room services) |  |  |

*: in interRAI MDS-HC 2.0
†: in interRAI-HC 9.1

| Supporting Information 3 | | | | | | | | | | | | | |
| --- | --- | --- | --- | --- | --- | --- | --- | --- | --- | --- | --- | --- | --- |
| Demographic information of community-dwelling older adults aged 65+ who was diagnosed with dementia | | | | | | | | | | | | | |
|  |  |  | **HK** | | |  | **NZ all** | | |  | **NZ Chinese** | | |
|  |  |  | **n (N=9796)** |  | **%** |  | **n (N=16725)** |  | **%** |  | **n (N=218)** |  | **%** |
| **Demographic Information of care-recipient** | | |  |  |  |  |  |  |  |  |  |  |  |
|  | Female |  | 5899 |  | 58.3 |  | 9564 |  | 57.2 |  | 124 |  | 56.9 |
|  | Age |  |  |  |  |  |  |  |  |  |  |  |  |
|  |  | 65-74 | 1148 |  | 13.2 |  | 2764 |  | 16.5 |  | 28 |  | 12.8 |
|  |  | 75-84 | 4436 |  | 49.8 |  | 7713 |  | 46.1 |  | 117 |  | 53.7 |
|  |  | 85+ | 4212 |  | 36.9 |  | 6248 |  | 37.4 |  | 73 |  | 33.5 |
|  | Marital Status | |  |  |  |  |  |  |  |  |  |  |  |
|  |  | Never married/Widowed/Separated/Divorced/Others | 5798 |  | 49.2 |  | 7999 |  | 47.8 |  | 111 |  | 50.9 |
|  |  | Married/Civil Union/Defacto | 3998 |  | 50.8 |  | 8721 |  | 52.1 |  | 107 |  | 49.1 |
|  | Living arrangement | |  |  |  |  |  |  |  |  |  |  |  |
|  |  | Alone | 624 |  | 15.1 |  | 5526 |  | 33 |  | 27 |  | 12.4 |
|  |  | With spouse/partner only | 888 |  | 21.2 |  | 7419 |  | 44.4 |  | 59 |  | 27.1 |
|  |  | with spouse/partner and others | 1521 |  | 26.6 |  | 1500 |  | 9 |  | 57 |  | 26.1 |
|  |  | with child | 6763 |  | 37.1 |  | 2280 |  | 13.6 |  | 75 |  | 34.4 |
|  | Prefer to live in elsewhere | | 6262 |  | 57.6 |  | 4994 |  | 29.9 |  | 72 |  | 33 |
|  |  |  |  |  |  |  |  |  |  |  |  |  |  |
| **Health and functional status of care-recipient** | | |  |  |  |  |  |  |  |  |  |  |  |
|  | Stay in hospital within 7days | | 1102 |  | 24.6 |  | 4474 |  | 26.8 |  | 73 |  | 33.5 |
|  | Hearing |  |  |  |  |  |  |  |  |  |  |  |  |
|  |  | Adequate | 3890 |  | 45.3 |  | 8815 |  | 52.7 |  | 125 |  | 57.3 |
|  |  | Minimal difficulty | 4229 |  | 41.8 |  | 4499 |  | 26.9 |  | 51 |  | 23.4 |
|  |  | Moderate to severe | 1677 |  | 13 |  | 3407 |  | 20.4 |  | 42 |  | 19.3 |
|  | Vision |  |  |  |  |  |  |  |  |  |  |  |  |
|  |  | Independent | 2932 |  | 36.9 |  | 12155 |  | 72.7 |  | 133 |  | 61 |
|  |  | Supervision | 5743 |  | 54.8 |  | 3261 |  | 19.5 |  | 66 |  | 30.3 |
|  |  | Limited to Total dependence | 1121 |  | 8.3 |  | 1305 |  | 7.8 |  | 19 |  | 8.7 |
|  | 3+ IADL items with difficulties | | 9672 |  | 98.2 |  | 15901 |  | 95.1 |  | 215 |  | 98.6 |
|  | ADL Hierarchy | |  |  |  |  |  |  |  |  |  |  |  |
|  |  | Independent | 3723 |  | 64.3 |  | 7392 |  | 44.2 |  | 80 |  | 36.7 |
|  |  | Supervision | 2163 |  | 20.9 |  | 4115 |  | 24.6 |  | 36 |  | 16.5 |
|  |  | Limited to Total dependence | 3910 |  | 14.8 |  | 5217 |  | 31.2 |  | 102 |  | 46.8 |
|  | Fall in last 90 days | | 3029 |  | 31.9 |  | 6356 |  | 38 |  | 91 |  | 41.7 |
|  | Comorbidity | |  |  |  |  |  |  |  |  |  |  |  |
|  |  | Dementia only | 1715 |  | 62.9 |  | 10008 |  | 59.8 |  | 136 |  | 62.4 |
|  |  | Stroke & Dementia | 637 |  | 21.6 |  | 1337 |  | 8 |  | 29 |  | 13.3 |
|  |  | CVD & Dementia | 4272 |  | 10.5 |  | 4274 |  | 25.6 |  | 33 |  | 15.1 |
|  |  | CVD, Stroke & Dementia | 3172 |  | 5.1 |  | 1106 |  | 6.6 |  | 20 |  | 9.2 |
| **Caregiver characteristics** | | |  |  |  |  |  |  |  |  |  |  |  |
|  | Primary caregiver relationship with care-recipient | |  |  |  |  |  |  |  |  |  |  |  |
|  |  | Child or child-in-law | 5503 |  | 57.2 |  | 7420 |  | 44.4 |  | 115 |  | 52.8 |
|  |  | Spouse/Partner/significant other | 2303 |  | 39.7 |  | 7653 |  | 45.8 |  | 87 |  | 39.9 |
|  |  | Parent/guardian/Sibling/Other relative or whanau/friends | 1990 |  | 3 |  | 1652 |  | 9.9 |  | 16 |  | 7.3 |
|  | Primary caregiver lives with care-recipient | | 4629 |  | 76.4 |  | 10691 |  | 63.9 |  | 176 |  | 80.7 |
|  | Primary caregiver provides IADL care | | 9171 |  | 96 |  | 14535 |  | 86.9 |  | 188 |  | 86.2 |
|  | Primary caregiver provides ADL care | | 7440 |  | 80.8 |  | 7979 |  | 47.7 |  | 132 |  | 60.6 |
|  | Primary caregiver provides more than 21hours of care in 7 days | | 2504 |  | 44 |  | 7192 |  | 43 |  |  |  |  |
| **Formal care services utilization of care-recipient** | | |  |  |  |  |  |  |  |  |  |  |  |
|  |  | Home services | 166 |  | 1.7 |  | 4157 |  | 24.9 |  | 115 |  | 52.8 |
|  |  | Visiting nurse | 600 |  | 6.1 |  | 963 |  | 5.8 |  | 13 |  | 6 |
|  |  | Allied-health services | 407 |  | 4.2 |  | 2074 |  | 12.4 |  | 59 |  | 27.1 |
|  |  | Hospital services | 5753 |  | 58.7 |  | 5934 |  | 35.5 |  | 86 |  | 39.4 |

| Supporting Information 4  Service utilization by ethnics or primary language use | | | | | |
| --- | --- | --- | --- | --- | --- |
|  |  | Visiting Nurse | Home-making | Allied health | Hospital service |
|  |  | N (%) | N (%) | N (%) | N (%) |
| **New Zealand** | | | | | |
|  | Chinese | 13 (6.0) | 31 (14.2) | 36 (16.5) | 74 (33.9) |
|  | Other Asians | 13 (6.1) | 27 (12.7) | 26 (12.3) | 76 (35.8) |
|  | European | 840 (5.9) | 3,813 (26.6) | 1,829 (12.8) | 5101 (35.6) |
|  | Maori | 43 (4.0) | 153 (14.2) | 76 (7.1) | 360 (33.4) |
|  | African | 6 (7.1) | 14 (16.7) | 15 (17.9) | 35 (41.7) |
|  | Pacific Peoples | 40 (5.8) | 90 (13.1) | 77 (11.2) | 242 (35.2) |
|  | Others | 7 (94.1) | 27 (22.9) | 14 (11.9) | 39 (33.1) |
|  |  |  |  |  |  |
| **Hong Kong** | | | | | |
|  | English speaker | 0 (0.0) | 1 (7.1) | 1 (7.1) | 9 (64.3) |
|  | Chinese speaker | 593 (6.1) | 164 (1.7) | 405 (4.2) | 5,717 (58.7) |
|  | Others | 7 (14.0) | 1 (2.0) | 1 (2.0) | 27 (54.0) |

|  | | | | | |
| --- | --- | --- | --- | --- | --- |
| Supporting information 5 | | | | | |
| Results of the logistic regression model including factors that were significant in both HK and NZ | | | | | |
|  |  |  |  |  |  |
|  |  |  | HK |  | NZ |
|  |  |  | AOR (95CI) |  | AOR (95CI) |
| **Demographic Information of care-recipient** | | |  |  |  |
|  | Female | | 1.01 (0.88, 1.16) |  | 0.88 (0.80, 0.98)* |
|  | Marital Status | |  |  |  |
|  |  | Never married/Widowed/Separated/Divorced/Other | 0.87 (0.71, 1.07) |  | 0.97 (0.77, 1.20) |
|  |  | Married/Civil Union/Defacto | Ref |  | Ref |
|  | Living arrangement | |  |  |  |
|  |  | Alone | Ref*** |  | Ref |
|  |  | With spouse/partner only | 0.87 (0.60, 1.25) |  | 1.38 (1.06, 1.80)* |
|  |  | With spouse/partner and others | 0.71 (0.50, 1.01) |  | 1.23 (0.94, 1.61) |
|  |  | With child | 0.58 (0.45, 0.75)*** |  | 0.99 (0.75, 1.31) |
|  | Perceived to be better-off living elsewhere | | 2.74 (2.39, 3.15)*** |  | 5.56 (4.99, 6.19)*** |
| **Health and functional status of care-recipient** | | |  |  |  |
|  | Hearing | |  |  |  |
|  |  | Adequate | Ref*** |  | Ref* |
|  |  | Minimal difficulty | 0.81 (0.71, 0.92)** |  | 0.93 (0.82, 1.04) |
|  |  | Moderate to severe difficulty | 1.19 (1.00, 1.40)* |  | 1.11 (0.98, 1.26) |
|  | Vision | |  |  |  |
|  |  | Adequate | Ref*** |  | Ref |
|  |  | Minimal difficulty | 0.99 (0.86, 1.13) |  | 0.98 (0.87, 1.11) |
|  |  | Moderate to severe difficulty | 0.67 (0.53, 0.84)*** |  | 1.01 (0.85, 1.19) |
|  | 3+ IADL items with difficulties | | 2.00 (0.90, 4.45) |  | 1.16 (0.83, 1.64) |
|  | ADL Hierarchy Scale | |  |  |  |
|  |  | Independent | Ref |  | Ref*** |
|  |  | Supervision | 0.99 (0.85, 1.16) |  | 1.41 (1.24, 1.61)*** |
|  |  | Limited to Total dependence | 1.06 (0.91, 1.23) |  | 1.26 (1.11, 1.44)*** |
|  | Fall history in 90 days | | 1.07 (0.94, 1.21) |  | 1.08 (0.97, 1.19) |
|  | Behavioral problems in past 3 days | | 1.61 (1.42, 1.83)*** |  | 1.51 (1.35, 1.68)*** |
|  | Mood problem in past 3 days | | 1.48 (1.27, 1.74)*** |  | 1.75 (1.55, 1.98)*** |
| **Caregiver characteristics** | | |  |  |  |
|  | Primary caregiver relationship with care-recipient | |  |  |  |
|  |  | Child or child-in-law | 1.57 (1.31, 1.88)*** |  | 1.19 (0.97, 1.46) |
|  |  | Spouse/Partner/significant other | 3.07 (2.44, 3.86)*** |  | 1.47 (1.10, 1.97)** |
|  |  | Parent/guardian/Sibling/Other relative or whanau/friends | Ref*** |  | Ref* |
|  | Primary caregiver lives with care-recipient | | 0.91 (0.77, 1.08) |  | 1.62 (1.27, 2.07)*** |
|  | Primary caregiver provides IADL care | | 1.18 (0.81, 1.71) |  | 0.85 (0.73, 0.99)* |
|  | Primary caregiver provides ADL care | | 2.97 (2.44, 3.62)*** |  | 1.21 (1.07, 1.36)** |
|  | Primary caregiver provides more than 21hours of care in 7days | | 1.57 (1.36, 1.82)*** |  | 1.38 (1.23, 1.55)*** |
| **Formal care services utilization of care-recipient** | | | |  |  |
|  | Home Service | |  |  |  |
|  | Visiting Nurse | |  |  |  |
|  | Allied-health Service | | 0.50 (0.34, 0.72)*** |  | 0.89 (0.77, 1.02) |
|  | Hospital Service | |  |  |  |
| ADL: activities of daily living; IADL: instrumental activities of daily living | | | | | |
| P-value of interaction value is denoted as *: <0.05; **:<0.01; ***:<0.001 | | | | | |

| Supporting information 6 | | | | | | | | | |
| --- | --- | --- | --- | --- | --- | --- | --- | --- | --- |
| Results of multigroup analysis with contributing factors that were significant in both HK and NZ | | | | | | | | | |
|  |  |  | Not using allied health services |  |  |  | Using allied health services |  |  |
|  |  |  | HK |  | NZ |  | HK |  | NZ |
|  |  |  | AOR (95CI) |  | AOR (95CI) |  | AOR (95CI) |  | AOR (95CI) |
| **Demographic Information of care-recipient** | | |  |  |  |  |  |  |  |
|  | Female | | 1.03 (0.89, 1.18) |  | 0.88 (0.79, 0.99)* |  | 0.49 (0.17, 1.37) |  | 0.90 (0.70, 1.16) |
|  | Marital Status | |  |  |  |  |  |  |  |
|  |  | Never married/Widowed/Separated/Divorced/Other | 0.86 (0.70, 1.06) |  | 1.05 (0.83, 1.34) |  | 2.02 (0.45, 9.04) |  | 0.59 (0.32, 1.09) |
|  |  | Married/Civil Union/Defacto | Ref |  | Ref |  | Ref |  | Ref |
|  | Living arrangement | |  |  |  |  |  |  |  |
|  |  | Alone | Ref |  | Ref |  | Ref |  | Ref |
|  |  | With spouse/partner only | 0.90 (0.62, 1.30) |  | 1.42 (1.06, 1.89)* |  | 0.24 (0.02, 3.33) |  | 1.19 (0.57, 2.48) |
|  |  | With spouse/partner and others | 0.72 (0.51, 1.03) |  | 1.16 (0.87, 1.55) |  | 0.22 (0.02, 3.01) |  | 1.61 (0.79, 3.30) |
|  |  | With child | 0.60 (0.46, 0.78)*** |  | 1.06 (0.78, 1.42) |  | 0.19 (0.03, 1.16) |  | 0.57 (0.27, 1.19) |
|  | Perceived to be better-off living elsewhere | | 2.71 (2.35, 3.11)*** |  | 5.68 (5.06, 6.38)*** |  | 4.18 (1.50, 11.67)** |  | 4.58 (3.36, 6.23)*** |
| **Health and functional status of care-recipient** | | | |  |  |  |  |  |  |
|  | Hearing | |  |  |  |  |  |  |  |
|  |  | Adequate | Ref*** |  | Ref* |  | Ref |  | Ref |
|  |  | Minimal difficulty | 0.81 (0.71, 0.92)** |  | 0.93 (0.82, 1.06) |  | 0.77 (0.29, 2.08) |  | 0.91 (0.68, 1.22) |
|  |  | Moderate to severe difficulty | 1.19 (1.00, 1.41)* |  | 1.17 (1.02, 1.33)* |  | 1.08 (0.28, 4.16) |  | 0.97 (0.71, 1.31) |
|  | Vision | |  |  |  |  |  |  |  |
|  |  | Adequate | Ref*** |  | Ref |  | Ref |  | Ref |
|  |  | Minimal difficulty | 1.00 (0.88, 1.15) |  | 1.00 (0.87, 1.14) |  | 0.48 (0.18, 1.25) |  | 0.95 (0.71, 1.26) |
|  |  | Moderate to severe difficulty | 0.66 (0.52, 0.83)*** |  | 1.09 (0.91, 1.32) |  | 1.12 (0.31, 3.96) |  | 0.70 (0.46, 1.06) |
|  | 3+ IADL items with difficulties | | 2.00 (0.90, 4.45) |  | 1.17 (0.82, 1.66) |  | NA |  | 0.55 (0.12, 2.65) |
|  | ADL Hierarchy Scale | |  |  |  |  |  |  |  |
|  |  | Independent | Ref |  | Ref*** |  | Ref |  |  |
|  |  | Supervision | 0.99 (0.85, 1.17) |  | 1.41 (1.23, 1.61)*** |  | 0.63 (0.17, 2.26) |  | 1.07 (0.70, 1.63) |
|  |  | Limited to Total dependence | 1.06 (0.91, 1.23) |  | 1.29 (1.13, 1.49)*** |  | 0.78 (0.25, 2.46) |  | 0.87 (0.58, 1.32) |
|  | Fall history in 90 days | | 1.06 (0.94, 1.21) |  | 1.08 (0.97, 1.21) |  | 1.31 (0.52, 3.30) |  | 1.04 (0.81, 1.35) |
|  | Behavioral problems in past 3 days | | 1.61 (1.42, 1.83)*** |  | 1.55 (1.37, 1.75)*** |  | 1.73 (0.60, 4.97) |  | 1.35 (1.04, 1.77)* |
|  | Mood problem in past 3 days | | 1.50 (1.28, 1.76)*** |  | 1.88 (1.65, 2.15)*** |  | 1.11 (0.31, 4.04) |  | 1.19 (0.86, 1.65) |
| **Caregiver characteristics** | | |  |  |  |  |  |  |  |
|  | Primary caregiver relationship with care-recipient | |  |  |  |  |  |  |  |
|  |  | Child or child-in-law | 1.59 (1.33, 1.92)*** |  | 1.15 (0.92, 1.45) |  | 0.73 (0.21, 2.56) |  | 1.32 (0.83, 2.10) |
|  |  | Spouse/Partner/significant other | 3.11 (2.46, 3.92)*** |  | 1.31 (0.95, 1.80) |  | 2.26 (0.58, 8.71) |  | 2.59 (1.23, 5.46)* |
|  |  | Parent/guardian/Sibling/Other relative or whanau/friends | Ref*** |  | Ref |  | Ref |  | Ref* |
|  | Primary caregiver lives with care-recipient | | 0.92 (0.77, 1.09) |  | 1.55 (1.19, 2.02)** |  | 0.57 (0.13, 2.45) |  | 2.32 (1.23, 4.37)** |
|  | Primary caregiver provides IADL care | | 1.15 (0.79, 1.68) |  | 0.86 (0.71, 1.03) |  | 5.05 (0.40, 64.47) |  | 0.85 (0.63, 1.15) |
|  | Primary caregiver provides ADL care | | 3.02 (2.47, 3.69)*** |  | 1.25 (1.09, 1.42)** |  | 1.04 (0.23, 4.78) |  | 1.02 (0.74, 1.39) |
|  | Primary caregiver provides more than 21hours of care in 7days | | 1.53 (1.32, 1.77)*** |  | 1.38 (1.22, 1.56)*** |  | 4.50 (1.60, 12.67)** |  | 1.26 (0.88, 1.78) |
| **Formal care services utilization of care-recipient** | | | |  |  |  |  |  |  |
|  | Home Service | |  |  |  |  |  |  |  |
|  | Visiting Nurse | |  |  |  |  |  |  |  |
|  | Allied-health Service | |  |  |  |  |  |  |  |
|  | Hospital Service | |  |  |  |  |  |  |  |
| ADL: activities of daily living; IADL: instrumental activities of daily living | | | | |  |  |  |  |  |
| P-value of interaction value is denoted as *: <0.05; **:<0.01; ***:<0.001 | | | | | |  |  |  |  |
